# Association between type 2 diabetes and site‐specific fracture risk: A systematic review and meta‐analysis of cohort studies including over 13 million participants

**DOI:** 10.1111/dme.70276

**Published:** 2026-03-13

**Authors:** Sara Naderpour, Clare Gillies, Paul O'Riordan, Noushin Fahimfar, Zahra Karimi, Malihe Khoramdad, Sonia Khavere, Monica Kundu, Kamlesh Khunti, Safoora Gharibzadeh

**Affiliations:** ^1^ Leicester Real World Evidence Unit, Diabetes Research Centre, College of Life Science University of Leicester Leicester UK; ^2^ Leicester Real World Evidence Unit, Diabetes Research Centre, Leicester General Hospital University of Leicester Leicester UK; ^3^ Department of Respiratory Sciences University of Leicester Leicester UK; ^4^ Department of Respiratory Medicine, Institute for Lung Health Glenfield Hospital Leicester UK; ^5^ Osteoporosis Research Center, Endocrinology and Metabolism Clinical Sciences Institute Tehran University of Medical Sciences Tehran Iran; ^6^ Department of Epidemiology and Biostatistics, School of Public Health Tehran University of Medical Sciences Tehran Iran; ^7^ Social Health Research Institute University of Social Welfare and Rehabilitation Sciences Tehran Iran; ^8^ Diabetes Research Centre, College of Life Science University of Leicester Leicester UK; ^9^ Department of Population Health Sciences University of Leicester Leicester UK

**Keywords:** bone fracture, fragility fracture, hip fracture, meta‐analysis, systematic review, type 2 diabetes

## Abstract

**Objective:**

This review aimed to quantify the association between Type 2 Diabetes (T2D) and the risk of fracture at various anatomical sites by synthesising data from cohort studies.

**Methods:**

A systematic search was conducted across Medline, Embase, CINAHL and Web of Science databases, from inception to 10 June 2025. We estimated pooled hazard ratios (HRs) with corresponding 95% confidence intervals using random‐effects models. This study is registered with PROSPERO (CRD42024548795).

**Results:**

This meta‐analysis of 22 studies, selected from 6534 screened studies, assessed a total of 13,074,868 individuals (2,644,443 people with T2D and 10,430,425 without T2D). People with T2D have a 25% increased risk of fractures (all anatomical sites) compared to individuals without T2D (HR: 1.25; 95% CI: 1.20 to 1.31). T2D was significantly associated with an increased risk of appendicular lower limb fractures (HR: 1.43; 95% CI: 1.30 to 1.57), upper limb fractures (HR: 1.29; 95% CI: 1.16 to 1.45), osteoporotic/fragility fractures (HR: 1.14; 95% CI: 1.02 to 1.28) and appendicular unspecified fractures (HR: 1.25; 95% CI: 1.05 to 1.48). Subgroup analyses indicated stronger associations in prospective studies. Women with T2D had a significantly higher fracture risk than men. Meta‐regression analyses showed that a higher percentage of women participants and a longer duration of T2D were associated with stronger associations between T2D and fracture risk, particularly for lower limb fractures.

**Conclusion:**

T2D is associated with an increased risk of fractures, especially in lower limbs (hip, ankle and foot). These findings highlight the importance of targeted fracture prevention strategies and site‐specific risk assessment for individuals with T2D. However, due to the heterogeneity among studies, caution is required in the interpretation of these findings.

## Graphical abstract

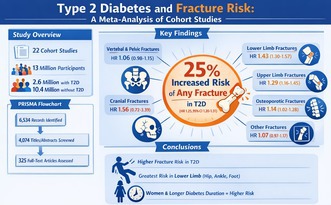

**What's new?**

**What is already known?**

- Previous systematic reviews and meta‐analyses have assessed fracture risk in individuals with T2D compared to people without T2D, but they were limited to a small number of skeletal sites.

**What this study has found?**

- This study provides a comprehensive assessment of fracture risk across more than 20 anatomical sites, offering a detailed view of skeletal vulnerability in individuals with T2D compared with individuals without T2D.
- Existing evidence indicates that individuals with T2D have a 25% higher risk of fractures across all anatomical sites compared to those without T2D, with the greatest increase observed at lower limb sites.

**What are the implications of the study?**

- Clinical guidelines should integrate site‐specific risk profiles for people with T2D.
- Future primary studies should explore how ethnicity influence fracture risk

## 1 INTRODUCTION

The global prevalence of Diabetes Mellitus (DM) continues to rise, with Type 2 Diabetes (T2D) accounting for approximately 96.0% of all cases worldwide.^1^, ^2^ According to the International Diabetes Federation (IDF) Atlas, 11th edition (2025), an estimated 589 million adults aged 20–79 were living with diabetes worldwide in 2024. By 2050, this figure is expected to increase to 853 million.^1^ In addition, T2D is the ninth leading cause of mortality, with over one million deaths annually worldwide, and the seventh leading cause of disability.^2^

The disease has a variety of complications associated with it, including cardiovascular disease, kidney failure, stroke, atherosclerosis and reduced physical function.^3^, ^4^ In particular, individuals with T2D may have altered calcium metabolism, elevated bone turnover rates and lower bone mineral density (BMD), factors that may collectively contribute to an increased risk of fractures.^5^

Despite advancements in diabetes therapeutics and management, diabetes‐related complications continue to pose a major healthcare problem.^6^ Evidence suggests an association between T2D and fracture risk; however, this relationship is not uniform across all skeletal sites,^7^, ^8^, ^9^ and the risk reported from earlier studies on individuals with T2D varies considerably. For instance, in some studies, hip fractures,^10^, ^11^, ^12^, ^13^, ^14^ vertebral fractures,^15^, ^16^, ^17^ wrist fractures^10^, ^18^ and any types of fractures^9^, ^19^ tend to be more prevalent in the population with T2D. Conversely, other studies have found no significant association between T2D and hip fractures,^18^, ^20^, ^21^, ^22^ vertebral fractures,^10^, ^13^, ^23^, ^24^ wrist fractures^20^, ^23^, ^24^, ^25^ or any fractures.^20^, ^24^

A previous study based on 25 cohort studies evaluated the association between both types of DM and fracture risk.^26^ The key limitation of this study was that the role of T2D was not assessed separately in relation to fracture risk. Additionally, the estimated fracture risk among people with T2D was not comprehensive, as it only considered five skeletal sites. Therefore, the present meta‐analysis of available cohort studies was conducted to determine the comprehensive fracture risk across more than 20 skeletal sites. Although total fractures include both low‐ and high‐trauma events, our primary analytical emphasis is on site‐specific and fragility‐relevant fractures. Total fractures are reported for completeness, but conclusions are based on anatomical sites where T2DM‐related skeletal and fall‐related mechanisms are most likely to act.

Understanding these differences is essential for developing precise, patient‐centred, targeted screening and prevention strategies that consider the unique fracture risks faced by individuals with T2D. Identifying skeletal sites that are more commonly affected in people with T2D, allows clinicians to prioritise screening at those locations, implement early interventions (e.g., fall prevention, bone‐strengthening therapies) and personalise monitoring protocols based on risk stratification‐ultimately improving patient outcomes and resource allocation.

Therefore, the aim of this systematic review and meta‐analysis is to combine evidence from cohort studies to determine the risk of fractures in adult participants with T2D compared to those without diabetes, across different fracture sites.

## 2 METHOD

This systematic review and meta‐analysis was reported based on the Preferred Reporting Items for Systematic Reviews and Meta‐Analyses (PRISMA)^27^ and Meta‐analysis Of Observational Studies in Epidemiology (MOOSE) guidelines^28^ (checklist provided in Tables S1 and S2). The study is registered with the International Prospective Register of Systematic Reviews (PROSPERO) under the registration number CRD42024548795.

### 2.1 Search strategy and inclusion criteria

A comprehensive systematic literature search was conducted across Medline, Embase, CINAHL and Web of Science databases to identify observational studies comparing fracture risk between people with and without diabetes, covering publications from inception to June 10, 2025. The search utilised keywords such as ‘Type 2 Diabetes’ and ‘Fracture’, with filters applied to limit the results to cohort studies in humans and articles published in English. The search strategy utilised for Medline is provided in supplementary information (Table S3). Additionally, the reference lists of potentially relevant articles were manually reviewed to identify any further eligible studies. We contacted the corresponding authors of the studies to request the full text when it was not available and also to obtain missing information needed for inclusion in the analyses.

All articles underwent an initial screening by SN, with ZK and SK acting as second reviewer and independently screened half the articles each. Any disagreements were resolved through discussion to ensure consistency. To be included in the meta‐analysis, studies had to meet the following inclusion criteria: (1) original observational cohort studies; (2) conducted in human adult populations (adults aged 18 years and above); (3) individuals with and without a diagnosis of T2D as the exposure; (4) incidence of fractures at any site as the outcome of interest.

Exclusion criteria were as follows: (1) studies involving T1D, gestational diabetes, prediabetes, or individuals at risk of diabetes, as well as studies stratifying participants based on glucose levels rather than overt diabetes mellitus; (2) studies that did not report results for T1D and T2D separately; (3) studies where diabetes was diagnosed after the occurrence of a fracture; (4) studies lacking data on fracture outcomes; (5) cross sectional, case control and intervention studies; (7) duplicate reports from the same cohort; (8)studies which do not include a control group; (9) review articles, conference abstracts, letters and commentaries; (9) studies with the population of children or adolescents.

### 2.2 Risk of bias and study quality

The quality of the cohort studies was assessed using the Newcastle–Ottawa Scale (NOS),^29^ a widely accepted tool for evaluating the quality of observational studies in meta‐analyses. This checklist consists of three categories of selection (four items with a total of four stars), comparability (one item with a total of two stars) and outcome (three items with a total of three stars) with a total of nine stars for assessment. Studies scoring between 7 and 9 stars were considered high‐quality. Quality assessments were independently conducted by two reviewers (SN and MK), and any inconsistencies were resolved by discussion. Publication bias was assessed by visual inspection of funnel plot and Egger test.^30^

### 2.3 Data extraction

Two authors (SN and ZK) independently extracted relevant data, with any discrepancies resolved through discussion. The extracted data included author, year of publication, geographical location of participants, mean age, proportion of men participants, study design, number of subjects with and without diabetes, number of fractures in the exposed and unexposed groups, follow‐up period, duration of diabetes and the type of fracture investigated.

We reported all fractures, which include any type of fractures identified in the included studies. Additionally, based on the variation in fracture sites reported in the studies, we classified fractures into five main anatomical categories to enable meaningful subgroup analysis: 1‐ Axial (Vertebral or Pelvic): This group includes clinical vertebral fractures, pelvis and lower back, thorax and upper back, clinical spine fractures, lumbar spine fractures. 2‐ Axial (Cranial): This group encompasses skull or face fractures. 3‐ Appendicular (Upper Limb): Fractures in this group include those of the upper limb, arm (proximal humerus and wrist), upper arm, proximal humerus, lower arm, radius, forearm and hand. 4‐ Appendicular (Lower Limb): This group includes fractures of the hip, lower limb, ankle and foot. 5‐ Appendicular unspecified: This category covers fractures not specifically identified as upper or lower limb but classified as non‐vertebral.

Additionally, some fracture categories reported in the primary studies did not align with anatomical groupings. Therefore, we classified them as follows:

Category 6‐ Osteoporotic and Fragility Fractures: This includes fractures specifically associated with osteoporosis or bone fragility. Category 7‐ Other Fractures: This category was used in some primary studies to refer to fractures not involving specific anatomical sites (e.g., hip, vertebrae), or where the definition of ‘Other fractures’ was not clearly specified.

### 2.4 Statistical analysis

The included primary studies reported three different measures of association: hazard ratio (HR) (17 studies), incidence rate ratio (IRR) (1 study) and relative risk (RR) (4 studies). Since most included studies reported HRs, we used HR across all three types of measures of association. The consideration of the RR as an estimate of the HR is predicated on the assumption of rare diseases, wherein RR serves as an approximation of HR when the incidence of fractures is low.^31^, ^32^ Furthermore, the incidence rate ratio (IRR) is regarded as an approximation of HR under conditions where incidence rates remain stable over time, in accordance with epidemiological principles pertaining to rare events.^33^ As such, the reported RRs and IRRs were considered appropriate approximations of a HR for the purpose of this meta‐analysis.

The heterogeneity among the studies included in the analysis was assessed using the *I* ^2^ statistic and Q statistics; *p*‐values less than 0.10 were deemed indicative of significant heterogeneity.^34^ Suggested interpretations for the *I* ^2^ were: low (0–40%), moderate (30–60%), substantial (50–90%) and considerable heterogeneity (75‐100%).^35^

All fitted meta‐analysis models assumed random effects to allow for between‐study heterogeneity and were fitted for all fractures and then by the seven anatomical subgroups defined. A sensitivity analysis was carried out, separating the seven subgroups into individual fracture sites, to assess if effect sizes were similar enough across the sites we combined to justify the clinical groupings. To explore potential methodological heterogeneity, we conducted additional meta‐analyses stratified by study design (prospective vs. retrospective) and, within retrospective studies, by setting (population‐based vs. hospital‐based). When only one study was present in a subgroup, meta‐analysis was not performed but the study results were retained in the forest plots to allow for comparison across subgroups.

Meta‐regression analyses were carried out to determine the influence of study‐level characteristics on the estimated fracture risk associated with T2D. Predictor variables included mean age, percentage of men participants, diabetes duration and study design. Meta‐regressions were only performed when data from at least three studies were available. Statistical analyses were carried out with Stata, version 18.0 (StataCorp. 2023. Stata Statistical Software: Release 18. College Station, TX: StataCorp LLC).

## 3 RESULTS

### 3.1 Literature search

**FIGURE 1:**
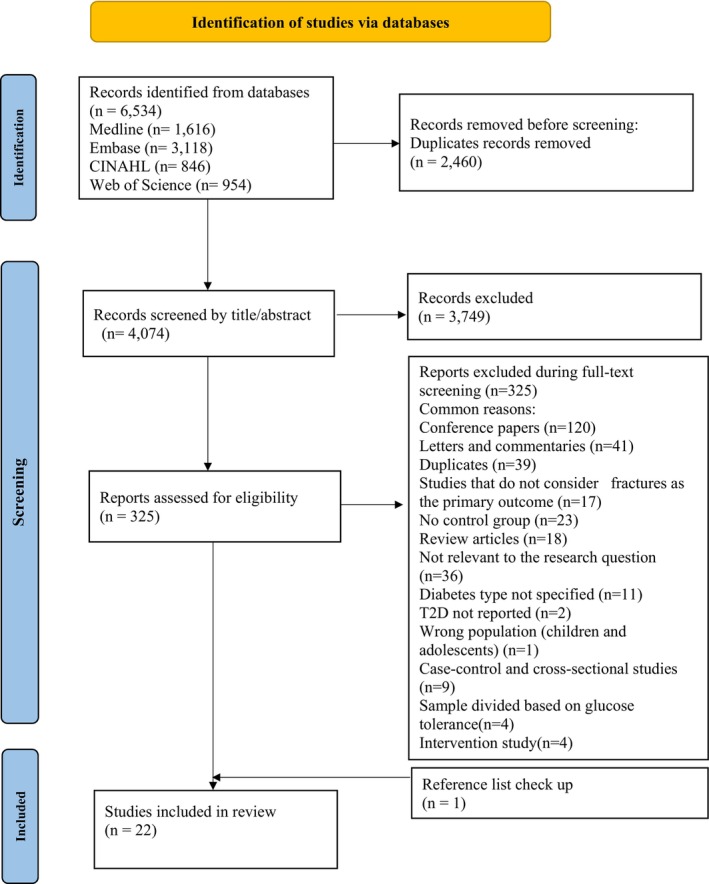
Flow chart: Flow diagram of the literature search and study selection.

The initial electronic search identified a total of 6534 articles. After removing duplicates, 4074 unique articles remained. Following a screening of titles and abstracts, 3749 articles were excluded. The remaining 325 studies were retrieved for full‐text evaluation. After further screening the full texts, 22 cohort studies were selected for inclusion in the final meta‐analysis. (Figure 1).

### 3.2 Baseline characteristics and quality assessment

Among 22 included studies, 14 were prospective cohort population studies,^8^, ^11^, ^25^, ^36^, ^37^, ^38^, ^39^, ^40^, ^41^, ^42^, ^43^, ^44^, ^45^, ^46^ six were retrospective cohort population‐based studies,^7^, ^9^, ^47^, ^48^, ^49^, ^50^ and two were retrospective cohort hospital‐based studies.^51^, ^52^ The studies were published between 2001 and 2024. The included studies comprised a total of 13,074,868 individuals; 2,644,443 with T2D among whom 143,046 fractures were reported and 10,430,425 individuals without T2D, with 997,752 fractures recorded. The mean age of participants ranged from 53.96 to 76.86 years, with most studies including both men and women participants. There was a wide geographical distribution in the included studies, 10 studies conducted in North America^9^, ^11^, ^38^, ^39^, ^40^, ^42^, ^43^, ^44^, ^45^, ^52^; eight in Europe^8^, ^25^, ^36^, ^46^, ^48^, ^49^, ^50^, ^51^; two in Asia^7^, ^47^ and two in Australia.^37^, ^41^ The duration of follow‐up ranged from 2.5 to 21 years. Most of the studies adjusted for key confounders such as age, sex, body mass index (BMI), smoking status, alcohol use, osteoporosis‐related factors and comorbidities (Table S4).

The quality assessment of the included studies by NOS indicated that most of the studies were of high quality, with scores ranging from 7 to 9, and three studies^14^, ^44^, ^53^ were categorised as moderate quality (Table S5).

### 3.3 Association between T2D and site‐specific fractures

#### 3.3.1 Total fracture risk estimation

**FIGURE 2:**
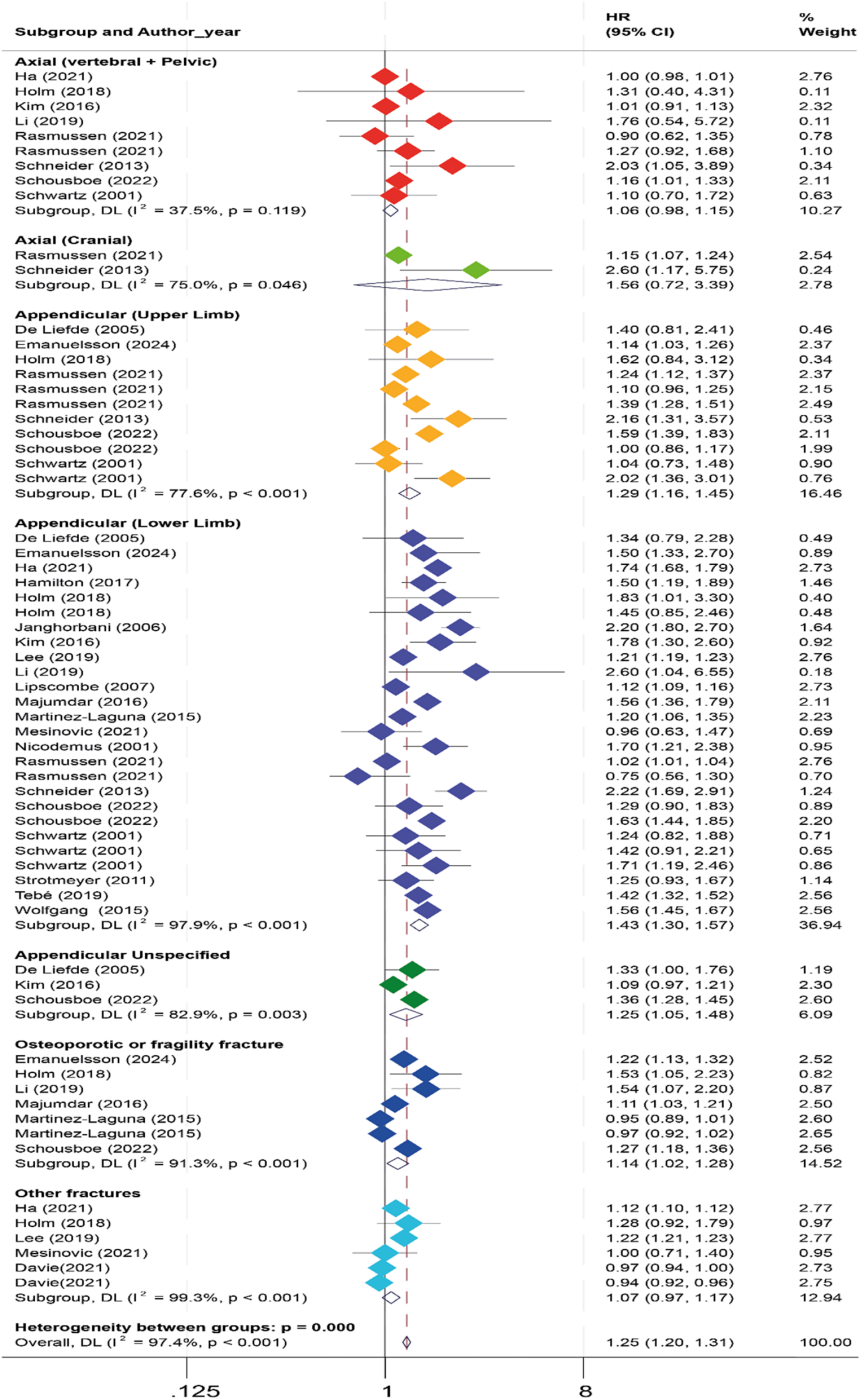
Forest plot of risk of fracture in various site fractures.

There was a 25% increased risk of fractures among individuals with T2D compared to those without. (HR 1.25, 95% CI: 1.20 to 1.31) (Figure 2). There was high heterogeneity (*I* ^2^ = 97.4%, *p* < 0.001) between studies. The results of the meta‐regression analyses to explore the potential sources of heterogeneity are reported in Tables 1 and 2. The analysis showed that a higher percentage of men participants was significantly associated with a lower estimated effect size (−0.003; 95% CI: −0.005 to −0.001; *p* = 0.007), that is studies with higher proportions of men participants reported lower hazard ratios for increased risk of fracture associated with T2D (Table 1). Additionally, increased mean diabetes duration at study level was associated with an increased risk of fractures associated with T2D (0.08; 95% CI: 0.002 to 0.15; *p* = 0.046) (Table 1).

**TABLE 1:** Meta‐regression analyses for all fracture sites.

Study level variable	Subset	*N* studies	Coefficient^a^ (95% CI)	*p*‐value
*Fracture risk (total)*				
Men %	–	22	**−0.003 (−0.005, −0.001)**	**0.007**
Mean age	–	22	−0.003 (−0.01, 0.008)	0.571
Continent	Asia (reference)	–	–	–
	Europe	8	0.06 (−0.32, 0.46)	0.734
	North America	10	0.04 (−0.31, 0.40)	0.798
	Australia	2	–	
Publication year		22	−0.009 (−0.02, 0.002)	0.127
Mean follow‐up (year)		18	−0.009 (−0.02, 0.004)	0.161
Diabetes duration		4	**0.08 (0.002, 0.15)**	**0.046**
Study design	Retrospective cohort hospital‐based (reference)	–	–	–
	Retrospective cohort population‐based	6	−0.10 (−0.26, 0.05)	0.189
	Prospective cohort population‐based	14	0.004 (−0.27, 0.27)	0.973

*Note*: Bold values indicate statistically significant association *p* < 0.05.

^a^ Coefficients from the meta‐regression analysis indicate the estimated change in the effect size (i.e., estimated HR for increased risk of fracture associated with T2D for a one unit increase in the predictor).

**TABLE 2:** Meta‐regression analyses stratified by fracture categories.

Predictor	Categories of fracture	*N* studies	Coefficient^a^ (95% CI)	*p*‐value
*Fracture risk*				
Men (%)	Axial (vertebral + pelvic)	9	−0.003 (−0.008,0.001)	0.084
	Axial (cranial)	–	–	–
	Appendicular (upper Limb)	11	−0.002 (−0.010,0.007)	0.713
	Appendicular (lower Limb)	26	**−0.005 (−0.008, −0.001)**	**0.012**
	Appendicular Unspecified i.e., upper or lower	3	−0.007 (−0.03,0.01)	0.178
	Osteoporotic or fragility fracture	7	−0.003 (−0.010,0.003)	0.235
	Other fractures	5	0.001 (−0.005,0.007)	0.650
Mean age	Axial (vertebral + pelvic)	9	0.006 (−0.01,0.02)	0.428
	Axial (cranial)	–	–	–
	Appendicular (upper limb)	11	0.002 (−0.03, 0.04)	0.889
	Appendicular (lower limb)	26	−0.02 (−0.041, 0.001)	0.055
	Appendicular unspecified i.e., upper or lower	3	0.02 (−0.68, 0.73)	0.737
	Osteoporotic or fragility fracture	7	−0.002 (−0.08, 0.08)	0.953
	Other fractures	5	0.003 (−0.01, 0.02)	0.598
Publication year	Axial (vertebral + pelvic)	9	0.0004 (−0.02, 0.02)	0.973
	Axial (cranial)	–	–	–
	Appendicular (upper limb)	11	−0.006 (−0.03, 0.02)	0.609
	Appendicular (lower limb)	26	−0.007 (−0.02, 0.01)	0.477
	Appendicular unspecified i.e., upper or lower	3	0.002 (−0.25, 0.25)	0.937
	Osteoporotic or fragility fracture	7	**0.031 (0.005, 0.057)**	**0.025**
	Other fractures	5	−0.10 (−0.22, −0.01)	0.081
Mean follow‐up time	Axial (vertebral + pelvic)	8	0.008 (−0.01, 0.03)	0.378
	Axial (cranial)	–	–	–
	Appendicular (upper limb)	9	−0.006 (−0.04,0.03)	0.689
	Appendicular (lower limb)	22	−0.016 (−0.04, 0.01)	0.252
	Appendicular unspecified i.e., upper or lower	3	0.08 (−0.22, 0.40)	0.177
	Osteoporotic or fragility fracture	4	0.03 (−0.34, 0.41)	0.752
	Other fractures	5	−0.025 (−0.05, 0.008)	0.108
Diabetes duration	Axial (vertebral + pelvic)	1	–	–
	Axial (cranial)	0	–	–
	Appendicular (upper limb)	2	–	–
	Appendicular (lower limb)	6	0.05 (−0.02, 0.13)	0.116
	Appendicular unspecified i.e., upper or lower	0	–	–
	Osteoporotic or fragility fracture	1	–	–
	Other fractures	0	–	–

*Note*: Bold values indicate statistically significant association *p* < 0.05.

^a^ Coefficients from the meta‐regression analysis indicate the estimated change in the effect size (i.e., estimated HR for increased risk of fracture associated with T2D for a one unit increase in the predictor).

Subgroup analyses by study design for all types of fracture showed no difference in pooled results between prospective and retrospective studies, or by setting (Figures S1–S4).

Additionally, sex‐specific subgroup analysis demonstrated that women with T2D had a significantly higher fracture risk than men (Figure S5).

#### 3.3.2 Axial (vertebral + pelvic) fractures

A total of 153,641 fractures were reported in 8 studies of this category of fracture, which involved a study population of 7,524,575 individuals. As shown in Figure 2, the pooled HR was 1.06 [95% CI: 0.98 to 1.15], indicating no significant association between fracture risk at these sites for individuals with T2D. This group of studies had limited heterogeneity between studies (*I* ^2^ = 37.5%, *p* = 0.119).

Subgroup analyses by study design for axial (vertebral + pelvic) fractures showed no difference in pooled results between prospective and retrospective studies (Figures S1 and S2).

#### 3.3.3 Appendicular (lower limb) fractures

Twenty‐one studies involving a total population of 12,233,572 people reported 92,898 fractures (Figure 2). We found a statistically significant positive association between T2D and lower limb fracture incidence (pooled HR = 1.43, 95% CI: 1.30 to 1.57). Heterogeneity was high (*I* ^2^ = 97.9%, *p* < 0.001); thus, we conducted meta‐regression analysis, which illustrated that the percentage of men participants in a study was significantly and inversely associated with the association between T2D and fracture risk in this category (−0.005; 95% CI: −0.008 to −0.001; *p* = 0.012), indicating that studies with a higher proportion of men had smaller effect sizes when assessing the association between T2D and fracture risk. This indicates that the impact of T2D on the risk of lower limb fractures may be greater in women (Table 2).

Subgroup analyses for lower limb fractures showed no difference in pooled results between prospective and retrospective studies, or by setting (Figures S1–S4).

#### 3.3.4 Osteoporotic or fragility fractures

A total of 37,085 fractures were reported in six studies, with a study population of 607,932 individuals. As shown in Figure 2, there was a 14% (HR 1.14, 95% CI: 1.02 to 1.28) significantly increased risk of fragility fractures with high heterogeneity (*I* ^2^ = 91.3%, *p* < 0.001). Meta‐regression analysis revealed a significant and positive association between the year of publication and risk estimates (0.031; 95% CI: 0.005 to 0.057; *p* = 0.025) (Table 2).

Subgroup analyses by study design for osteoporotic or fragility fractures showed similar pooled estimates, although the association was statistically significant only in retrospective studies (Figures S2–S4).

#### 3.3.5 Other fractures

A total of 814,630 fractures were reported in five studies, with a study population of 10,274,199 individuals. As shown in Figure 2, the HR is 1.07 (95% CI: 0.97 to 1.17), showing no significant association between fracture risk and T2D in the category of other fractures. High heterogeneity is observed (*I* ^2^ = 99.3%, *p* < 0.001). Meta‐regression showed a non‐significant negative association between publication year and mean follow‐up duration with estimated effect size (−0.10; 95% CI: −0.22 to −0.01; *p* = 0.08) and (−0.025; 95% CI: −0.05 to 0.008; *p* = 0.108) respectively (Table 2).

Similarly, subgroup analyses by study design for category of other fractures showed no difference in pooled results between prospective and retrospective studies (Figures S1 and S2).

#### 3.3.6 Axial (cranial) fractures

Overall, 2261 fractures were reported in two studies, with a study population of 828,553 individuals. As shown in Figure 2, the HR is 1.56 (95% CI: 0.72 to 3.39), suggesting no significant potential increase in fracture risk. A significant level of heterogeneity was detected (*I* ^2^ = 75%, *p* = 0.046), and meta‐regression did not identify any statistically substantial covariates that explain this variation (Table 2).

Subgroup analysis by study design for axial (cranial) fractures was limited, with only one study available for each of the prospective and retrospective categories (Figures S1 and S2).

#### 3.3.7 Appendicular (upper limb) fractures

In total, seven studies involving 27,339 fractures and a population of 1,128,083 individuals reported the association between T2D and upper limb fracture (Figure 2). Compared with non‐DM, individuals with T2D had a higher risk of upper limb fracture (pooled HR: 1.29; 95% CI: 1.16 to 1.45). Evidence of significant heterogeneity was identified (*I* ^2^ = 77.6%, *p* < 0.001). Therefore, we did meta‐regression analysis, which had no effect on the result (Table 2).

Subgroup analysis by study design for appendicular (upper limb) fractures showed no difference in pooled results between prospective and retrospective studies. The hospital‐based group had only a single study (Figure S4).

#### 3.3.8 Appendicular (unspecified) fractures

A total of 12,944 fractures were reported in three studies, with a study population of 138,223 individuals. As shown in Figure 2, the HR is 1.25 [95% CI: 1.05 to 1.48], showing a statistically significant increase in fracture risk. High heterogeneity is observed (*I* ^2^ = 82.9%, *p* < 0.003); then, we did meta‐regression analysis, which showed no statistically significant association between covariates and estimated study effect sizes (Table 2).

Subgroup analysis by study design for appendicular (unspecified) fractures was limited, with only two retrospective studies and one prospective study available and no data were available from hospital‐based retrospective studies for this fracture category (Figures S1, S2 and S4).

### 3.4 Publication bias

From the review of the funnel plot and according to Egger's test (*p*‐value for Egger: 0.0002), there was evidence of publication bias. The funnel plot of the 22 studies included in the metanalysis is provided in figure supplementary 6 (Figure S6). The sensitivity analyses separating the seven clinical subgroups further (Figure S6) found similar pooled effect sizes for the sites we had combined, justifying our primary analysis.

## 4 DISCUSSION

This systematic review of over 13 million people demonstrates that T2D is associated with a higher risk of various fracture types, including total fractures and appendicular fractures in the upper limb, lower limb and unspecified areas, as well as osteoporotic or fragility fractures. However, we did not find a significant link between T2D and the risk of axial fractures, which encompass both vertebral and pelvic fractures, as well as cranial fractures, and the category of other fractures. Importantly, our subgroup analyses by study design and setting reveal meaningful differences. Prospective studies generally reported higher hazard ratios than retrospective studies, suggesting that study design may influence observed effect sizes, potentially due to more accurate exposure and outcome ascertainment, as well as more consistent adjustment for confounders. While our meta‐analysis provides pooled HR by incorporating time‐to‐event data, most prior studies have reported risk ratios (RR). Despite these methodological distinctions, the direction and impact of our findings align with those of earlier studies.^26^, ^38^, ^53^, ^54^, ^55^

The pathophysiological reason for this relationship is still unclear. Despite normal or even elevated BMD in individuals with T2D, this paradox indicates that bone quality, rather than bone quantity, is effective in fracture susceptibility in these participants.^56^ It appears that the association between T2D and bone disease occurs through complex pathways, including the insulin growth factor (IGF) system, accumulation of advanced glycation end products (AGEs) in bone collagen, microangiopathy and increased bone marrow fat content. Collectively, these alterations result in impaired bone quality and increased fragility.^57^, ^58^, ^59^ Moreover, microvascular complications, such as neuropathy, retinopathy and muscle weakness, along with insulin therapy, which can cause hypoglycaemias, substantially increase the risk of falls and finally fractures in individuals with T2D.^60^

It is noteworthy that previous studies have reported conflicting results regarding the effects of T2D on bone fracture risk. For example, Oei et al. showed no significant relationship between adequate or inadequately controlled T2D and the risk of hip fracture in people with T2D in comparison with non‐diabetic individuals.^18^ Differences in the classification of the participants based on subdivision of blood sugar control may be the reason for this discrepancy. Similarly, Gerdhem et al. reported an increased risk of any fracture or forearm fracture in T2D people when compared with non‐diabetic individuals, although these increases were not statistically significant.^24^ One potential explanation is that the cohort comprised merely 39 women who experienced any fracture and four women who sustained a forearm fracture among a total of 74 individuals diagnosed with T2D. Consequently, the limited sample size and the low incidence of fracture events likely contributed to diminished statistical power.

Although high heterogeneity was observed across several pooled analyses, our meta‐regression analyses provided essential insights into the potential sources of this variability. We identified that the fracture risk associated with T2D was significantly modified by the proportion of men participants and the duration of diabetes. Studies with a higher percentage of men participants reported lower fracture risks. In contrast, studies with longer diabetes duration showed higher fracture risks, suggesting that sex distribution and disease chronicity are important contributors to variability across studies.

Further stratified meta‐regression by fracture category revealed that a higher proportion of men was particularly associated with a lower risk of lower limb fractures, indicating sex‐specific differences in anatomical site vulnerability. Additionally, publication year emerged as a significant factor, with more recent studies tending to report higher risks for osteoporotic or fragility fractures, likely reflecting advances in diagnostic capabilities, osteoporosis awareness and improvements in statistical methodologies over time, such as more consistent adjustment for confounding variables.

Despite identifying several contributing factors, a substantial proportion of heterogeneity remains unexplained, likely due to variations in unmeasured confounders such as comorbidities, medication use, glycaemic control or fracture definitions. This underlines the complexity of synthesising observational data in this context and highlights the need for individual participant data meta‐analyses in the future.

In addition to diabetes duration and complications, accumulating evidence indicates that fracture risk in individuals with T2D may vary according to the class of glucose‐lowering medication used. Evidence has demonstrated an increased fracture risk associated with thiazolidinediones, particularly among women, an effect attributed to suppression of osteoblast activity and altered bone turnover.^61^, ^62^ Insulin therapy has also been associated with a higher fracture risk in several population‐based cohort studies, likely reflecting both greater disease severity and an increased risk of hypoglycaemia‐related falls.^63^, ^64^ In contrast, metformin has generally shown neutral or potentially protective effects on skeletal health,^10^ while incretin‐based therapies and sodium–glucose cotransporter‐2 inhibitors have not demonstrated consistent increases in fracture risk in large cardiovascular and renal outcome trials, despite early safety concerns with specific agents.^65^ Antidiabetic medication use may therefore represent an important unmeasured confounder contributing to residual heterogeneity and should be addressed in future individual participant data meta‐analyses.

This review has some strengths. First, we examined fracture risk across a wide range of anatomical sites—more than 20—offering a comprehensive view of skeletal vulnerability in individuals with T2D. This site‐specific approach enabled us to identify patterns of risk that might be overlooked in analyses limited to hip or vertebral fractures alone. Second, by using HRs as the primary measure of the association between T2D and bone fracture, our time‐to‐event analysis confirmed a temporal association, thereby strengthening the reliability of our estimates. Third, a large number of included studies with a population of over 13 million enhances the statistical power and generalisability of our findings.

Despite these strengths, several limitations of this study should be noted. Due to limited resources, we had to exclude studies not published in English. Also, considerable heterogeneity was observed across multiple analyses, particularly in estimates of total fractures and some site‐specific categories. Despite the valuable effect of meta‐regression in identifying influential factors, such as sex ratio and duration of diabetes, unexplained heterogeneity may have affected the interpretation of the pooled results. Moreover, retrospective cohort studies may contribute to the recall and selection biases observed in this study. The adjusted models of the included studies were different; these variables may have been very important in causing fractures. Finally, publication bias and unavailability of individual data were two inherent limitations of any meta‐analysis.

## 5 CONCLUSION

In summary, this systematic review and meta‐analysis highlight evidence that T2D is associated with an increased risk of upper limb, lower limb, non‐vertebral, osteoporotic or fragility fractures, and other fractures compared with non‐diabetics. Moreover, the risk varies by the location of fracture, and this association is most pronounced in the lower limb (hip, ankle and foot) region. In contrast, vertebral and skull or facial fractures were non‐significant. These results warrant cautious interpretation, as substantial heterogeneity exists across study populations and methodological approaches. At the same time, future primary studies should investigate the impact of ethnicity and on fracture risk—factors that could not be adequately assessed in the current study due to substantial missing data. Additionally, the roles of diabetic complications, antidiabetic therapies and fall prevention strategies in reducing fracture burden among individuals with T2D warrant further evaluation.

These findings emphasise the necessity of including fracture risk assessment in diabetes prevention particularly for women and individuals with long‐standing T2D. Clinicians should consider site‐specific risks and individualised prevention strategies.

## AUTHOR CONTRIBUTIONS

SGH, CG and SN developed the idea of the study. SN developed the search strategy and conducted the database searches. CG and SGH supervised the search strategy. SN, ZK and SK screened the studies. SN and MK assessed the quality of the studies. SN and ZK undertook data extraction. SN and MLK carried out the statistical analysis, which was supervised by CG and SGH. CG also reviewed the analysis and code for accuracy and critically appraised the results. PO and NF contributed to defining the fracture categories based on anatomical site. SN interpreted the findings, with supervision from CG and SGH. SN drafted the manuscript. SGH, CG, KK, PO and NF critically reviewed the manuscript, and SN revised it for final submission. All authors have approved the final draft of the manuscript. SN accepted full responsibility for the work and the conduct of the study, had access to the data and controlled the decision to publish. The corresponding author attests that all listed authors meet authorship criteria and that no others meeting the criteria have been omitted.

## FUNDING INFORMATION

This research was funded by the National Institute for Health and Care Research (NIHR) Leicester Biomedical Research Centre (BRC).

## CONFLICT OF INTEREST STATEMENT

Sara Naderpour, Clare Gillies, Paul O'Riordan, Noushin Fahimfar, Zahra Karimi, Malihe Khoramdad, Sonia Khavere, Monica Kundu and Safoora Gharibzadeh declare that they have no conflict of interest. Prof Kamlesh Khunti has acted as a consultant, speaker or received grants for investigator‐initiated studies for Abbott, Astra Zeneca, Bayer, Novo Nordisk, Sanofi‐Aventis, Servier, Lilly and Merck Sharp & Dohme, Boehringer Ingelheim, Oramed Pharmaceuticals, Pfizer, Roche, Daiichi‐Sankyo, Applied Therapeutics, Embecta and Nestle Health Science.

## Supporting information


Data S1:
