# Supplementary material for: Association between type 2 diabetes and site‐specific fracture risk: A systematic review and meta‐analysis of cohort studies including over 13 million participants
Source: Diabet Med. 2026 Mar 13;43(5):e70276. doi: 10.1111/dme.70276 (PMC13074118; doi:10.1111/dme.70276)

**Supplementary Materials**

**Table S1: PRISMA 2020 Checklist**

| **Section and Topic** | **Item #** | **Checklist item** | **Location where item is reported** |
| --- | --- | --- | --- |
| **TITLE** | | |  |
| Title | 1 | Identify the report as a systematic review. | 0 |
| **ABSTRACT** | | |  |
| Abstract | 2 | See the PRISMA 2020 for Abstracts checklist. | 2 |
| **INTRODUCTION** | | |  |
| Rationale | 3 | Describe the rationale for the review in the context of existing knowledge. | 1, 2 |
| Objectives | 4 | Provide an explicit statement of the objective(s) or question(s) the review addresses. | 2 |
| **METHODS** | | |  |
| Eligibility criteria | 5 | Specify the inclusion and exclusion criteria for the review and how studies were grouped for the syntheses. | 2, 3 |
| Information sources | 6 | Specify all databases, registers, websites, organisations, reference lists and other sources searched or consulted to identify studies. Specify the date when each source was last searched or consulted. | 2 |
| Search strategy | 7 | Present the full search strategies for all databases, registers and websites, including any filters and limits used. | 2, S3 |
| Selection process | 8 | Specify the methods used to decide whether a study met the inclusion criteria of the review, including how many reviewers screened each record and each report retrieved, whether they worked independently, and if applicable, details of automation tools used in the process. | 3, 4 |
| Data collection process | 9 | Specify the methods used to collect data from reports, including how many reviewers collected data from each report, whether they worked independently, any processes for obtaining or confirming data from study investigators, and if applicable, details of automation tools used in the process. | 2-4 |
| Data items | 10a | List and define all outcomes for which data were sought. Specify whether all results that were compatible with each outcome domain in each study were sought (e.g. for all measures, time points, analyses), and if not, the methods used to decide which results to collect. | 3, 4 |
|  | 10b | List and define all other variables for which data were sought (e.g. participant and intervention characteristics, funding sources). Describe any assumptions made about any missing or unclear information. | 3, 4 |
| Study risk of bias assessment | 11 | Specify the methods used to assess risk of bias in the included studies, including details of the tool(s) used, how many reviewers assessed each study and whether they worked independently, and if applicable, details of automation tools used in the process. | 3 |
| Effect measures | 12 | Specify for each outcome the effect measure(s) (e.g. risk ratio, mean difference) used in the synthesis or presentation of results. | 4 |
| Synthesis methods | 13a | Describe the processes used to decide which studies were eligible for each synthesis (e.g. tabulating the study intervention characteristics and comparing against the planned groups for each synthesis (item #5)). | 2-4 |
|  | 13b | Describe any methods required to prepare the data for presentation or synthesis, such as handling of missing summary statistics, or data conversions. | 3, 4 |
|  | 13c | Describe any methods used to tabulate or visually display results of individual studies and syntheses. | 4 |
|  | 13d | Describe any methods used to synthesize results and provide a rationale for the choice(s). If meta-analysis was performed, describe the model(s), method(s) to identify the presence and extent of statistical heterogeneity, and software package(s) used. | 4 |
|  | 13e | Describe any methods used to explore possible causes of heterogeneity among study results (e.g. subgroup analysis, meta-regression). | 4, 5 |
|  | 13f | Describe any sensitivity analyses conducted to assess robustness of the synthesized results. | - |
| Reporting bias assessment | 14 | Describe any methods used to assess risk of bias due to missing results in a synthesis (arising from reporting biases). | 4 |
| Certainty assessment | 15 | Describe any methods used to assess certainty (or confidence) in the body of evidence for an outcome. | 4, 5 |
| **RESULTS** | | |  |
| Study selection | 16a | Describe the results of the search and selection process, from the number of records identified in the search to the number of studies included in the review, ideally using a flow diagram. | 5 |
|  | 16b | Cite studies that might appear to meet the inclusion criteria, but which were excluded, and explain why they were excluded. | 5 |
| Study characteristics | 17 | Cite each included study and present its characteristics. | 5 |
| Risk of bias in studies | 18 | Present assessments of risk of bias for each included study. | 6-8 |
| Results of individual studies | 19 | For all outcomes, present, for each study: (a) summary statistics for each group (where appropriate) and (b) an effect estimate and its precision (e.g. confidence/credible interval), ideally using structured tables or plots. | 6-8 |
| Results of syntheses | 20a | For each synthesis, briefly summarise the characteristics and risk of bias among contributing studies. | 6-8 |
|  | 20b | Present results of all statistical syntheses conducted. If meta-analysis was done, present for each the summary estimate and its precision (e.g. confidence/credible interval) and measures of statistical heterogeneity. If comparing groups, describe the direction of the effect. | 6-8 |
|  | 20c | Present results of all investigations of possible causes of heterogeneity among study results. | 6-8 |
|  | 20d | Present results of all sensitivity analyses conducted to assess the robustness of the synthesized results. | - |
| Reporting biases | 21 | Present assessments of risk of bias due to missing results (arising from reporting biases) for each synthesis assessed. | 6-8 |
| Certainty of evidence | 22 | Present assessments of certainty (or confidence) in the body of evidence for each outcome assessed. | 6-8 |
| **DISCUSSION** | | |  |
| Discussion | 23a | Provide a general interpretation of the results in the context of other evidence. | 8-9 |
|  | 23b | Discuss any limitations of the evidence included in the review. | 10 |
|  | 23c | Discuss any limitations of the review processes used. | 10 |
|  | 23d | Discuss implications of the results for practice, policy, and future research. | 11 |
| **OTHER INFORMATION** | | |  |
| Registration and protocol | 24a | Provide registration information for the review, including register name and registration number, or state that the review was not registered. | 2 |
|  | 24b | Indicate where the review protocol can be accessed, or state that a protocol was not prepared. | 2 |
|  | 24c | Describe and explain any amendments to information provided at registration or in the protocol. | NA |
| Support | 25 | Describe sources of financial or non-financial support for the review, and the role of the funders or sponsors in the review. | 12 |
| Competing interests | 26 | Declare any competing interests of review authors. | 12 |
| Availability of data, code and other materials | 27 | Report which of the following are publicly available and where they can be found: template data collection forms; data extracted from included studies; data used for all analyses; analytic code; any other materials used in the review. | 12-Supplementry |

*From:*  Page MJ, McKenzie JE, Bossuyt PM, Boutron I, Hoffmann TC, Mulrow CD, et al. The PRISMA 2020 statement: an updated guideline for reporting systematic reviews. BMJ 2021;372:n71. doi: 10.1136/bmj.n71. This work is licensed under CC BY 4.0. To view a copy of this license, visit

**Table S2 : MOOSE Checklist**

**MOOSE (Meta-analyses Of Observational Studies in Epidemiology) Checklist**

A reporting checklist for Authors, Editors, and Reviewers of Meta-analyses of Observational Studies. You must report the page number in your manuscript where you consider each of the items listed in this checklist. If you have not included this information, either revise your manuscript accordingly before submitting or note N/A.

| **Reporting Criteria** | **Reported (Yes/No)** | | | **Reported on Page No.** | | |
| --- | --- | --- | --- | --- | --- | --- |
| **Reporting of Background** |  | | |  | | |
| Problem definition |  | Yes |  |  | 1 |  |
| Hypothesis statement |  | Yes |  |  | 1 |  |
| Description of Study Outcome(s) |  | Yes |  |  | 1 |  |
| Type of exposure or intervention used |  | Yes |  |  | 1,2 |  |
| Type of study design used |  | Yes |  |  | 1,2 |  |
| Study population |  | Yes |  |  | 2 |  |
| **Reporting of Search Strategy** |  | | |  | | |
| Qualifications of searchers (eg, librarians  and investigators) | Yes | | | 2 | | |
| Search strategy, including time period  included in the synthesis and keywords | Yes | | | 2-S3 | | |
| Effort to include all available studies,  including contact with authors | Yes | | |  | | |
|  |  |  |  |  | 2 |  |
| Databases and registries searched |  | Yes |  |  | 2 |  |
| Search software used, name and  version, including special features used (eg, explosion) | Yes | | | 2-S3 | | |
| Use of hand searching (eg, reference  lists of obtained articles) | Yes | | | 2-Fig 1 | | |
| List of citations located and those  excluded, including justification | Yes | | | 2, 5, Fig 1 | | |
| Method for addressing articles  published in languages other than English | Yes | | | 2, S3 | | |
| Method of handling abstracts and  unpublished studies | Yes | | | 2, 3 | | |
| Description of any contact with authors |  | Yes |  |  | 2 |  |
| **Reporting of Methods** |  | | |  | | |
| Description of relevance or appropriateness of studies assembled for  assessing the hypothesis to be tested | Yes | | | 2, 3 | | |
| Rationale for the selection and coding of data (eg, sound clinical principles or  convenience) | Yes | | | 4, 5 | | |
| Documentation of how data were classified and coded (eg, multiple raters,  blinding, and interrater reliability) | Yes | | | 4, 5 | | |
| Assessment of confounding (eg, comparability of cases and controls in  studies where appropriate | Yes | | | 5, S4(Table 1) | | |

| **Reporting Criteria** | **Reported (Yes/No)** | | | **Reported on Page No.** | | |
| --- | --- | --- | --- | --- | --- | --- |
| Assessment of study quality, including blinding of quality assessors; stratification or regression on possible  predictors of study results | Yes | | | 3,5, 6, S5 | | |
| Assessment of heterogeneity |  | Yes |  |  | 4, 5, Fig 2 |  |
| Description of statistical methods (eg,  complete description of fixed or random effects models, justification of whether the chosen models account for predictors of study results, dose-response models, or cumulative meta-analysis) in sufficient  detail to be replicated | Yes | | | 4, 5 | | |
| Provision of appropriate tables and  graphics | Yes | | | F1-2 ,T2-3, S3-5 | | |
| **Reporting of Results** |  | | |  | | |
| Table giving descriptive information for  each study included | Yes | | | S4(Table 1) | | |
| Results of sensitivity testing (eg,  subgroup analysis) | Yes | | | 6-8, Table 2-3 | | |
| Indication of statistical uncertainty of  findings | Yes | | | 10, 11 | | |
| **Reporting of Discussion** |  | | |  | | |
| Quantitative assessment of bias (eg,  publication bias) | Yes | | | 8, 9 | | |
| Justification for exclusion (eg, exclusion  of non–English-language citations) | Yes | | | 10 | | |
| Assessment of quality of included studies |  | Yes |  |  | 5, 6-S5 |  |
| **Reporting of Conclusions** |  | | |  | | |
| Consideration of alternative explanations  for observed results | Yes | | | 8-10 | | |
| Generalization of the conclusions (ie, appropriate for the data presented and  within the domain of the literature review) | Yes | | | 10, 11 | | |
| Guidelines for future research |  | Yes |  |  | 11 |  |
| Disclosure of funding source |  | Yes |  |  | 12 |  |

**Once you have completed this checklist, please save a copy and upload it as part of your submission. DO NOT include this checklist as part of the main manuscript document. It must be uploaded as a separate file.**

**Table S3 -Search Strategy**

**Ovid MEDLINE**

| **#** | **Search** | **Result** |
| --- | --- | --- |
| 1 | (diabetes adj3 (type 2 or type ii or type two)).mp. |  |
| 2 | exp Diabetes Mellitus, Type 2/ |  |
| 3 | (NIDDM or T2DM or T2D).mp. |  |
| 4 | non-insulin-dependent diabetes mellitus.mp. |  |
| 5 | adult-onset Diabetes Mellitus.mp. |  |
| 6 | stable diabetes mellitus.mp. |  |
| 7 | 1 OR 2 OR 3 OR 4 OR 5 OR 6 |  |
|  |  |  |
| 8 | fractur*.ti,ab. |  |
| 9 | exp Fractures, Bone/ |  |
| 10 | (bone* adj5 (injur* or break* or broke*)).ti,ab. |  |
| 11 | compression fracture*.mp. |  |
| 12 | Spiral Fracture*.mp. |  |
| 13 | Torsion Fracture*.mp. |  |
| 14 | 8 OR 9 OR 10 OR 11 OR 12 OR 13 |  |
|  |  |  |
| 15 | gestational diabetes.mp. or exp Diabetes, Gestational/ |  |
| 16 | prediabetes.mp. or exp Prediabetic State/ |  |
| 17 | 15 OR 16 |  |
|  |  |  |
| 18 | (Control* adj3 (trial* or study or studies)).mp |  |
| 19 | Randomi?ed.ab. |  |
| 20 | exp randomised controlled trial/ |  |
| 21 | randomly.ab. |  |
| 22 | trial.ti |  |
| 23 | placebo.ab. |  |
| 24 | clinical trials as topic/ |  |
| 25 | controlled clinical trial.pt. |  |
| 26 | 18 OR 19 OR 20 OR 21 OR 22 OR 23 OR 24 OR 25 |  |
|  |  |  |
| 27 | 7 AND 14 |  |
| 28 | 27 NOT 17 |  |
| 29 | 28 NOT 26 |  |
| 30 | limit 29 to English language | 1602 |
| Update | limit 30 to dt=20240607-20250610 | 166 |

**EMBASE**

| **#** | **Search** | **Result** |
| --- | --- | --- |
| 1 | (diabetes adj3 (type 2 or type ii or type two)).mp. |  |
| 2 | Exp non-insulin-dependent diabetes mellitus/ |  |
| 3 | (NIDDM or T2DM or T2D).mp. |  |
| 4 | adult-onset Diabetes Mellitus.mp. |  |
| 5 | stable diabetes mellitus.mp. |  |
| 6 | 1 OR 2 OR 3 OR 4 OR 5 |  |
|  |  |  |
| 7 | fractur*.ti,ab. |  |
| 8 | exp fracture/ |  |
| 9 | (bone* adj5 (injur* or break* or broke*)).ti,ab. |  |
| 10 | compression fracture*.mp. |  |
| 11 | Spiral Fracture*.mp. |  |
| 12 | Torsion Fracture*.mp. |  |
| 13 | 7 OR 8 OR 9 OR 10 OR 11 OR 12 |  |
|  |  |  |
| 14 | exp gestational diabetes/ |  |
| 15 | Diabetes, Gestational.mp. |  |
| 16 | Prediabetic State/ or prediabet*.mp. |  |
| 17 | 14 OR 15 OR 16 |  |
|  |  |  |
| 18 | (Control* adj3 (trial* or study or studies)).mp |  |
| 19 | Randomi?ed.ab. |  |
| 20 | exp randomised controlled trial/ |  |
| 21 | randomly.ab. |  |
| 22 | trial.ti |  |
| 23 | placebo.ab. |  |
| 24 | clinical trials as topic/ |  |
| 25 | exp controlled clinical trial/ |  |
| 26 | 18 OR 19 OR 20 OR 21 OR 22 OR 23 OR 24 OR 25 |  |
|  |  |  |
| 27 | 6 AND 13 |  |
| 28 | 27 NOT 17 |  |
| 29 | 28 NOT 26 |  |
| 30 | limit 29 to English language | 3079 |
| Update | limit 30 to dc=20240607-20250610 | 298 |

**CINAHL**

| **#** | **Search** | **Result** |
| --- | --- | --- |
| S1 | (MH "Diabetes Mellitus, Type 2") |  |
| S2 | diabetes N3 (type 2 or type ii or type two) |  |
| S3 | NIDDM or T2DM or T2D |  |
| S4 | non-insulin-dependent diabetes mellitus |  |
| S5 | adult-onset Diabetes Mellitus |  |
| S6 | stable diabetes mellitus |  |
| S7 | S1 OR S2 OR S3 OR S4 OR S5 OR S6 |  |
|  |  |  |
| S8 | fractur* or (MH "Fractures+") |  |
| S9 | "Bone Fracture" |  |
| S10 | bone* N5 (injur* or break* or broke*) |  |
| S11 | (MH "Fractures, Compression+") |  |
| S12 | "Spiral Fracture*" |  |
| S13 | "Torsion Fracture*" |  |
| S14 | S8 OR S9 OR S10 OR S11 OR S12 OR S13 |  |
|  |  |  |
| S15 | "prediabet*" OR (MH "Prediabetic State") |  |
| S16 | (MM "Diabetes Mellitus, Gestational") OR "gestational diabetes" |  |
| S17 | S15 OR S16 |  |
|  |  |  |
| S18 | (Control* N3 (trial* or study or studies)) |  |
| S19 | Randomis* |  |
| S20 | Randomiz* |  |
| S21 | (MH "Randomized Controlled Trials+") |  |
| S22 | AB randomly |  |
| S23 | TI trial |  |
| S24 | AB placebo |  |
| S25 | (MH "Clinical Trials+") OR "controlled clinical trial" |  |
| S26 | S18 OR S19 OR S20 OR S21 OR S22 OR S23 OR S24 OR S25 |  |
|  |  |  |
| S27 | S7 AND S14 |  |
| S28 | S27 NOT S17 |  |
| S29 | S28 NOT S26 |  |
| S30 | limit 29 to English language | 557 |
| Update | S30 AND EM 20240607–20250610 | 328 |

**Web of Science**

| **#** | **Search** | **Result** |
| --- | --- | --- |
| 1 | diabetes NEAR/3 ("type 2" or "type ii" or "type two") (Topic) |  |
| 2 | "type 2 diabetes mellitus" OR "NIDDM" OR "T2DM" OR "T2D" OR "non-insulin-dependent diabetes mellitus" OR "adult-onset diabetes mellitus" OR "stable diabetes mellitus" (Topic) |  |
|  | (TS=(diabetes NEAR/3 ("type 2" or "type ii" or "type two") (Topic))) OR TS=("type 2 diabetes mellitus" OR "NIDDM" OR "T2DM" OR "T2D" OR "non-insulin-dependent diabetes mellitus" OR "adult-onset diabetes mellitus" OR "stable diabetes mellitus" (Topic) ) |  |
| 3 | #1 OR #2 |  |
| 4 | bone* NEAR/5 (injur* or break* or broke*) (Topic) |  |
| 5 | "Compression fracture*" OR "Spiral Fracture*" OR "Torsion Fracture*" (Topic) |  |
| 6 | "Bone fracture*" (Topic) |  |
| 7 | fractur* (Title) |  |
| 8 | #4 OR #5 OR #6 OR #7 |  |
|  | (((TS=(bone* NEAR/5 (injur* or break* or broke*) )) OR TS=("Bone fracture*" )) OR TS=("Compression fracture*" OR "Spiral Fracture*" OR "Torsion Fracture*" )) OR TI=(fractur* ) |  |
| 9 | (gestation* NEAR/3 diabet*) OR prediabet* OR "GDM" OR (diabet* and pregnan*) (Topic) |  |
| 10 | #3 NOT #9 |  |
| 11 | Control* NEAR/3 (trial* or study or studies) (Topic) OR "randomi?ed controlled trial" OR placebo OR "clinical trial*" OR "control* clinical trial*"  (Topic) OR random*  (Title) |  |
| 12 | #10 NOT #11 |  |
|  | ((TI=((TS=(diabetes NEAR/3 ("type 2" or "type ii" or "type two") (Topic))) OR TS=("type 2 diabetes mellitus" OR "NIDDM" OR "T2DM" OR "T2D" OR "non-insulin-dependent diabetes mellitus" OR "adult-onset diabetes mellitus" OR "stable diabetes mellitus" (Topic) ))) NOT TI=((gestation* NEAR/3 diabet*) OR prediabet* OR "GDM" OR (diabet* and pregnan*) (Topic) )) NOT TI=(Control* NEAR/3 (trial* or study or studies) (Topic) OR "randomi?ed controlled trial" OR placebo OR "clinical trial*" OR "control* clinical trial*" (Topic) OR random* (Title) ) |  |
| 13 | #3 AND #8 |  |
|  | (ALL=((TS=(diabetes NEAR/3 ("type 2" or "type ii" or "type two") (Topic))) OR TS=("type 2 diabetes mellitus" OR "NIDDM" OR "T2DM" OR "T2D" OR "non-insulin-dependent diabetes mellitus" OR "adult-onset diabetes mellitus" OR "stable diabetes mellitus" (Topic) ))) AND ALL=((((TS=(bone* NEAR/5 (injur* or break* or broke*) )) OR TS=("Bone fracture*" )) OR TS=("Compression fracture*" OR "Spiral Fracture*" OR "Torsion Fracture*" )) OR TI=(fractur* )) |  |
| 14 | #13 NOT #9 |  |
|  | ((ALL=((TS=(diabetes NEAR/3 ("type 2" or "type ii" or "type two") (Topic))) OR TS=("type 2 diabetes mellitus" OR "NIDDM" OR "T2DM" OR "T2D" OR "non-insulin-dependent diabetes mellitus" OR "adult-onset diabetes mellitus" OR "stable diabetes mellitus" (Topic) ))) AND ALL=((((TS=(bone* NEAR/5 (injur* or break* or broke*) )) OR TS=("Bone fracture*" )) OR TS=("Compression fracture*" OR "Spiral Fracture*" OR "Torsion Fracture*" )) OR TI=(fractur* ))) NOT ALL=((gestation* NEAR/3 diabet*) OR prediabet* OR "GDM" OR (diabet* and pregnan*) (Topic) ) |  |
| 15 | #14 NOT #11 |  |
|  | (((ALL=((TS=(diabetes NEAR/3 ("type 2" or "type ii" or "type two") (Topic))) OR TS=("type 2 diabetes mellitus" OR "NIDDM" OR "T2DM" OR "T2D" OR "non-insulin-dependent diabetes mellitus" OR "adult-onset diabetes mellitus" OR "stable diabetes mellitus" (Topic) ))) AND ALL=((((TS=(bone* NEAR/5 (injur* or break* or broke*) )) OR TS=("Bone fracture*" )) OR TS=("Compression fracture*" OR "Spiral Fracture*" OR "Torsion Fracture*" )) OR TI=(fractur* ))) NOT ALL=((gestation* NEAR/3 diabet*) OR prediabet* OR "GDM" OR (diabet* and pregnan*) (Topic) )) NOT ALL=(Control* NEAR/3 (trial* or study or studies) (Topic) OR "randomi?ed controlled trial" OR placebo OR "clinical trial*" OR "control* clinical trial*" (Topic) OR random* (Title) ) |  |
| 16 | #14 NOT #11 and English (Languages) | 1,889 |
|  | TS=((diabetes NEAR/3 ("type 2" OR "type ii" OR "type two")  OR "type 2 diabetes mellitus"  OR NIDDM  OR T2DM  OR T2D  OR "non-insulin-dependent diabetes mellitus"  OR "adult-onset diabetes mellitus"  OR "stable diabetes mellitus")  AND  ((bone* NEAR/5 (injur* OR break* OR broke*))  OR "bone fracture*"  OR "compression fracture*"  OR "spiral fracture*"  OR "torsion fracture*"  OR fractur*)  NOT  ((gestation* NEAR/3 diabet*)  OR prediabet*  OR GDM  OR (diabet* AND pregnan*))  NOT  ((Control* NEAR/3 (trial* OR study OR studies))  OR "randomi?ed controlled trial"  OR placebo  OR "clinical trial*"  OR "control* clinical trial*"  OR random*))  AND LD=(2024-06-07/2025-06-10) |  |
| Update | LD=(2024-06-07/2025-06-10) | 209 |

**Table S4: The characteristics of identified studies and involved participants**

| **Author’s surname** | **Year** | **Country** | **Sex** | **Age (Mean± SD^b^)** | **No of Fracture/With T2D** | **No of Fracture/Without DM** | **Study Design** | **Follow-up year (mean± SD)** | **Data source** | **Adjustment** |
| --- | --- | --- | --- | --- | --- | --- | --- | --- | --- | --- |
| Martinez-Laguna(9) | 2015 | Spain | Both | 62.5± 11.8 | 2612 / 58483 | 4996 / 113448 | Prospective Cohort Population based | Med: 2.63(2.93) | Sistema d’Informació per al Desenvolupament de la Investigació en Atenció Primària (SIDIAP) | Body mass index(BMI), previous fracture, corticosteroid use, cardiovascular disease, kidney disease​ |
| Kim(8) | 2016 | Korean | Both | 63.9±8.3 | 1651 / 17110 | 2906 / 34220 | Retrospective Cohort population based | 6 years | NHS–National Sample Cohort (NHIS-NSC) | Age, household income, osteoporosis, and comorbidities |
| de Liefde(26) | 2005 | Netherlands | Both | 69.4±9 | 166 / 792 | 1,025 / 5,863 | Prospective Cohort Population based | 6.8 ±2.3 | The Rotterdam Study (GP and hospital records) | Age, gender, BMI, smoking, serum creatinine, visual acuity |
| Janghorbani(39) | 2006 | USA | F | 56.3±9.3 | 125 / 8348 | 1255 / 101343 | Prospective Cohort Population based | 20 ±4.3 | **The Nurses’ Health Study (NHS)** | Age, BMI, physical activity, menopausal status and estrogen use, smoking and daily intake of calcium, vitamin D, and protein |
| Nicodemus(43) | 2001 | USA | F | 61.5±5.9 | 38 / 1682 | 452 / 30377 | Prospective Cohort Population based | 11 years | Iowa Women’s Health Study (IWHS) | Age, smoking (former, current, never), estrogen use (former, current, never), BMI, and waist-to-hip ratio |
| Schousboe(10) | 2022 | USA | Both | 64.4±5.7 | 3698 / 8676 | 29527 / 71562 | Retrospective Cohort population based | 9.0 ±5.0 | Population-based DXA registry with linkage to Manitoba administrative health databases | Age, sex, history of fracture, femoral neck BMD, BMI, parental history of hip fracture, smoking status, high alcohol use, diagnosis of rheumatoid arthritis, socioeconomic status, and use of glucocorticoid, aromatase inhibitor, and fracture prevention medication. |
| Schneider(44) | 2013 | USA | Both | 54.1±5.7 | NA / 1195 | NA / 13340 | Prospective Cohort Population based | Med: 20 | The Atherosclerosis Risk in Communities (ARIC) Study | Age, sex, race/study centre, BMI, sports activity tertile, alcohol consumption, cigarette smoking, glucocorticoid or antidepressant use, thiazide diuretic use |
| Rasmussen(50) | 2021 | Denmark | Both | 58.2±5.6 | 5604 / 407009 | 5200 / 407009 | Retrospective Cohort population based | 21 years | Danish National Patient Registry linked to the Danish National Prescription Registry (nationwide administrative health registries) | Age, sex, diabetic complications, history of alcohol abuse, use of SSRIs, opioids, and anxiolytics |
| Strotmeyer(46) | 2011 | USA | Both | 72.8±5.4 | 65 / 918 | 269 / 2588 | Prospective Cohort Population based | 10.9 ± 4.6 | Cardiovascular Health Study (CHS) | Age, sex, race, BMI, subclinical PAD (ankle-arm index <0.9), current smoking, current drinking, 15 ft walk time |
| Mesinovic(42) | 2021 | Australia | Both | 76.8±11.7 | 74 / 471 | 209 / 1234 | Prospective Cohort Population based | 8.8 ± 3.6 | Concord Health and Ageing in Men Project (CHAMP) | Age, past year falls, living alone, physical activity , chronic pain, depression, number of comorbidities, obesity (body fat percentage and BMI), visual acuity, contrast sensitivity, cognitive status, and BMD. |
| Li(40) | 2019 | Canada | Both | 65.1±11.2 | 44 / 138 | 685 / 3011 | Prospective Cohort Population based | 9.2 ± 4.5 | Canadian Multicentre Osteoporosis Study (CaMos) | Age, sex, study centre, fasting insulin level, BMI, family history of fractures, previous falls, use of osteoporosis medication, use of pioglitazone or rosiglitazone, smoking, alcohol intake, and BMD femoral neck T-scores. |
| Hamilton(38) | 2017 | Australia | Both | 64±5.3 | 96 / 1291 | 328 / 5159 | Prospective Cohort Population based | 14.1 ± 5.9 | Fremantle Diabetes Study Phase I (FDS1) | Age, sex |
| Schwartz(45) | 2001 | USA | F | 71.7±11 | 189 / 657 | 2267 / 8997 | Prospective Cohort Population based | 9.4 ± 2 | Study of Osteoporotic Fractures (SOF) | Age, BMI, BMD, height, height loss since age 25, contrast sensitivity, walking speed, alcohol consumption, resting pulse, maternal hip fracture history, time spent on feet per day, use of long-acting benzodiazepines, and calcium intake. |
| Majumdar(41) | 2016 | Canada | F | 64.3±11 | 1097 / 8840 | 5320 / 49098 | Prospective Cohort Population based | 7 years | Population-based DXA registry with linkage to Manitoba administrative health databases | Age, BMI, previous fractures, FRAX score (with BMD), comorbidity, falls requiring hospitalisation, osteoporosis therapy, insulin therapy. |
| Emanuelsson(37) | 2024 | Denmark | Both | 57 [47–67) | 2,396 / 6,432 | 14,666 / 110,028 | Prospective Cohort Population based | Med: 10(Range:0.1-43) | - **Copenhagen City Heart Study (CCHS)**   **-Copenhagen General Population Study (CGPS)**  **-UK Biobank** | Age, sex, BMI, current smoking, physical activity level, units of alcohol per week, time since last meal (for glucose), and menopausal status for women |
| Tebé(47) | 2019 | Spain | Both | 72.1±4.3 | 1424 / 44802 | 1893 / 81233 | Prospective Cohort Population based | Med: 8(Range:7.3-8) | SIDIAP database | Age, sex, previous major osteoporotic fracture, previous ischemic heart disease (IHD), previous cerebrovascular disease (CVD), previous nephropathy, corticoid prescriptions, calcium + vitamin D, and anti-osteoporosis drugs. |
| Ha(48) | 2021 | South Korea | Both | 53.9±10 | 87,578 / 506,208 | 149,662 / 6040158 | Retrospective Cohort population based | 6.7 ± 1.7 | **Korean National Health Insurance Service (NHIS) database** | Age, sex, BMI, smoking history, alcohol consumption, regular exercise, income level, hypertension, hyperlipidemia, fasting glucose, and duration of diabetes. |
| Holm(52) | 2018 | Denmark | F | 61.1±11.6 | 133 / 229 | 2855 / 6056 | Retrospective Cohort hospital based | 5.8 years | **Clinical osteoporosis database** at **Copenhagen University Hospital Hvidovre** | Age, BMI, osteoporosis status, exercise level, medication use, comorbidities, and other osteoporosis risk factors. |
| Lipscombe(12) | 2007 | Canada | M | 73.6±5.9 | 7583 / 197412 | 14684 / 401400 | Prospective Cohort Population based | 6.1 ± 2.6 | **Ontario population-wide administrative health databases** | Age, comorbidity, prior stroke, visual impairment, neuropathy, amputation, and various medications affecting BMD and fall risk |
| Lee(53) | 2019 | USA | M | 71.9±6.6 | 25840 / 900402 | 56905 / 1897907 | Retrospective Cohort hospital based | 10 years | **• Veterans Health Administration (VHA) administrative databases**  **• Linked Centers for Medicare and Medicaid Services (CMS) Medicare fee-for-service data** | Age, race, ethnicity, body mass index, alcohol and tobacco use, rheumatoid |
| Wolfgang(49) | 2015 | Germany | Both | 66.1±12.2 | 2633 / 299104 | 1227 / 299104 | Retrospective Cohort population based | 2.5 ± 3.04 | **German Disease Analyzer database** (IMS Health) | Age, sex, diabetologist care, depression, chronic kidney |
| Davie(51) | 2021 | UK | Both | 60.8±13.1 | NA / 174,244 | NA / 747,290 | Retrospective Cohort population based | 15 years | The Health Improvement Network (THIN) | Age, BMI, Townsend score, and year of type 2 diabetes diagnosis |

**Table S5: Quality scores of cohort studies using the Newcastle-Ottawa Scale.**

| **Study** | **Selection^1^** | | | | **Comparability^2^** | **Outcome^3^** | | | **Total Score** | **Quality** |
| --- | --- | --- | --- | --- | --- | --- | --- | --- | --- | --- |
|  | Representa tiveness of the Exposed Cohort | Selection of the non exposed cohort | Ascertainment of  exposure | Demonstration that outcome of interest was not present at start of  study | Comparability of Cohorts on the Basis of the Design or Analysis | Assessment of outcome | Follow up long enough | Adequacy of follow up |  |  |
| Martinez-Laguna 2015(14) | ⭐ | ⭐ | ⭐ | ⭐ | ⭐⭐ | ⭐ | ⭐ | ⭐ | 9 | - High |
| Kim 2016(13) | ⭐ | ⭐ | ⭐ | ⭐ | ⭐⭐ | ⭐ | ⭐ | - | 8 | - High |
| de Liefde 2005(31) | ⭐ | ⭐ | ⭐ | ⭐ | ⭐⭐ | ⭐ | ⭐ | ⭐ | 9 | - High |
| Janghorbani 2006(41) | - | ⭐ | ⭐ | ⭐ | ⭐⭐ | ⭐ | ⭐ | ⭐ | 8 | - High |
| Nicodemus 2001(44) | ⭐ | ⭐ | - | ⭐ | ⭐⭐ | - | ⭐ | ⭐ | 7 | - High |
| Schousboe 2022(15) | - | ⭐ | ⭐ | - | ⭐⭐ | ⭐ | ⭐ | - | 6 | - Moderate |
| Schneider 2013(45) | ⭐ | ⭐ | - | - | ⭐⭐ | ⭐ | ⭐ | ⭐ | 7 | - High |
| Rasmussen 2021(50) | - | ⭐ | ⭐ | - | ⭐⭐ | ⭐ | ⭐ | ⭐ | 7 | - High |
| Strotmeyer 2011(47) | - | ⭐ | ⭐ | ⭐ | ⭐⭐ | - | ⭐ | ⭐ | 7 | - High |
| Mesinovic 2021(3) | - | ⭐ | ⭐ | ⭐ | ⭐⭐ | ⭐ | ⭐ | ⭐ | 8 | - High |
| Li 2019(42) | ⭐ | ⭐ | ⭐ | - | ⭐⭐ | ⭐ | ⭐ | ⭐ | 8 | - High |
| Hamilton 2017(40) | ⭐ | ⭐ | ⭐ | ⭐ | ⭐⭐ | ⭐ | ⭐ | ⭐ | 9 | - High |
| Schwartz 2001 (46) | - | ⭐ | - | - | ⭐⭐ | ⭐ | ⭐ | ⭐ | 6 | - Moderate |
| Majumdar 2016 (43) | - | ⭐ | ⭐ | ⭐ | ⭐⭐ | ⭐ | ⭐ | ⭐ | 8 | - High |
| Emanuelsson 2024(39) | - | ⭐ | ⭐ | - | ⭐⭐ | ⭐ | ⭐ | ⭐ | 7 | - High |
| Tebé 2019(48) | ⭐ | ⭐ | ⭐ | ⭐ | ⭐⭐ | ⭐ | ⭐ | ⭐ | 9 | - High |
| Ha 2021(49) | - | ⭐ | ⭐ | ⭐ | ⭐⭐ | ⭐ | ⭐ | ⭐ | 8 | - High |
| Holm 2018 (52) | - | ⭐ | ⭐ | ⭐ | ⭐⭐ | ⭐ | ⭐ | ⭐ | 8 | - High |
| Lipscombe 2007(17) | - | ⭐ | ⭐ | ⭐ | ⭐⭐ | ⭐ | ⭐ | ⭐ | 8 | - High |
| Lee 2019(53) | - | ⭐ | ⭐ | ⭐ | ⭐⭐ | ⭐ | ⭐ | ⭐ | 8 | - High |
| Wolfgang (50) | ⭐ | ⭐ | ⭐ | ⭐ | ⭐⭐ | ⭐ | - | - | 7 | - High |
| Davie(62) | - | ⭐ | - | ⭐ | ⭐⭐ | - | ⭐ | ⭐ | 6 | - Moderate |

^1^ Selection (maximum 4 stars), ^2^ Comparability (maximum 2 stars), and ^3^ Outcome (maximum 3 stars)

**Fig. S1: Forest plot of risk of fracture in various site fractures in prospective studies**


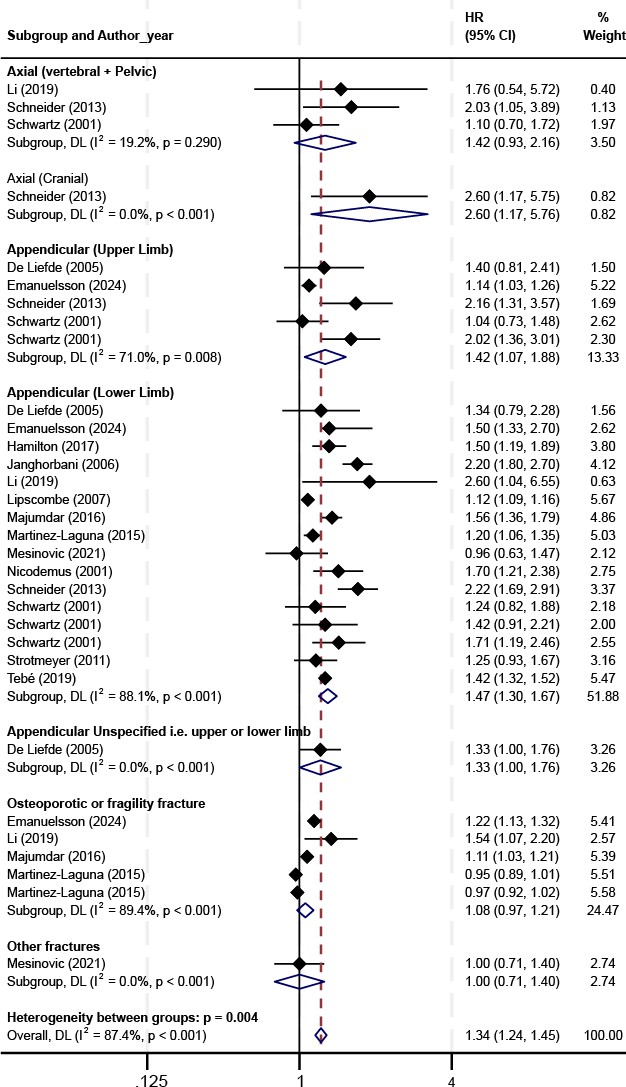


**Fig. S2: Forest plot of risk of fracture in various site fractures in retrospective studies**


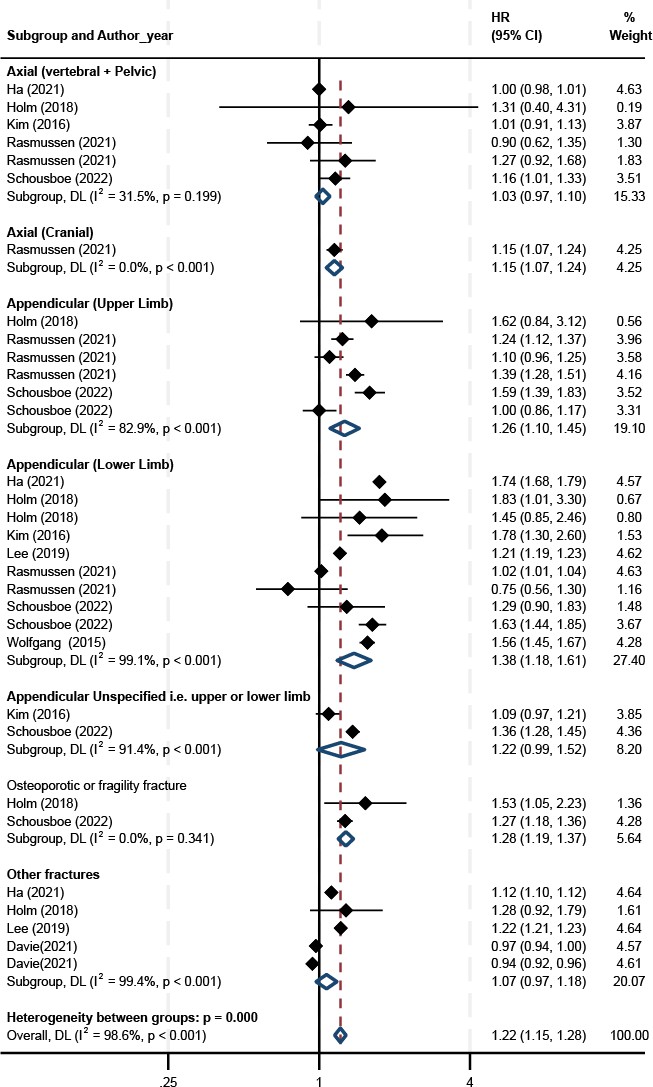


**Fig. S3: Forest plot of risk of fracture in various site fractures in retrospective (population based) studies**


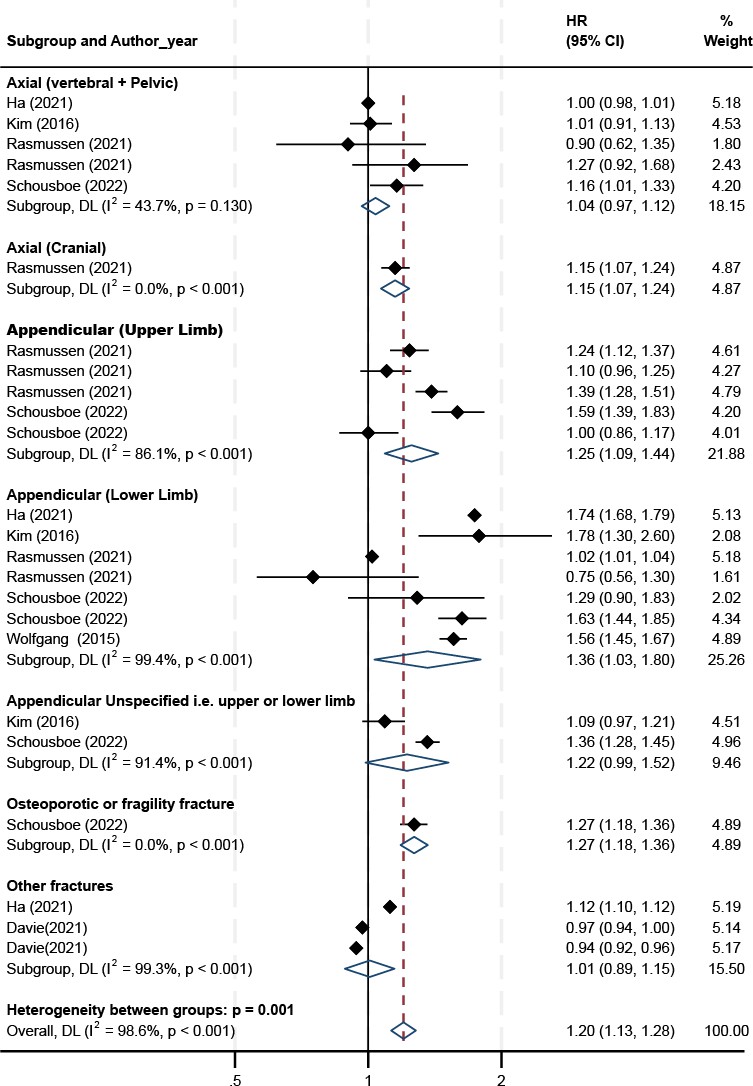


**Fig. S4: Forest plot of risk of fracture in various site fractures in retrospective (hospital based) studies**


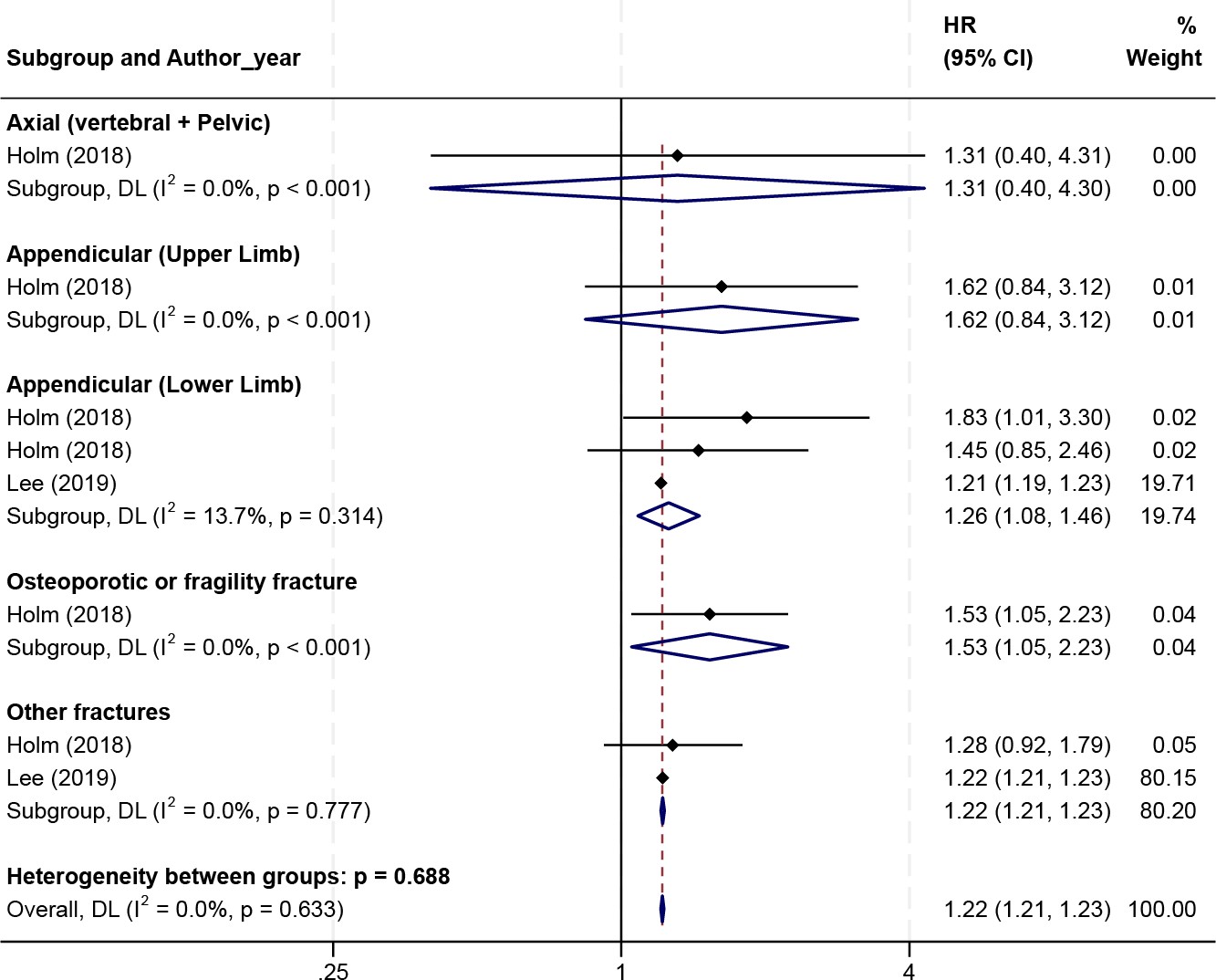


**Fig. S5: Forest plot of risk of fracture by sex**


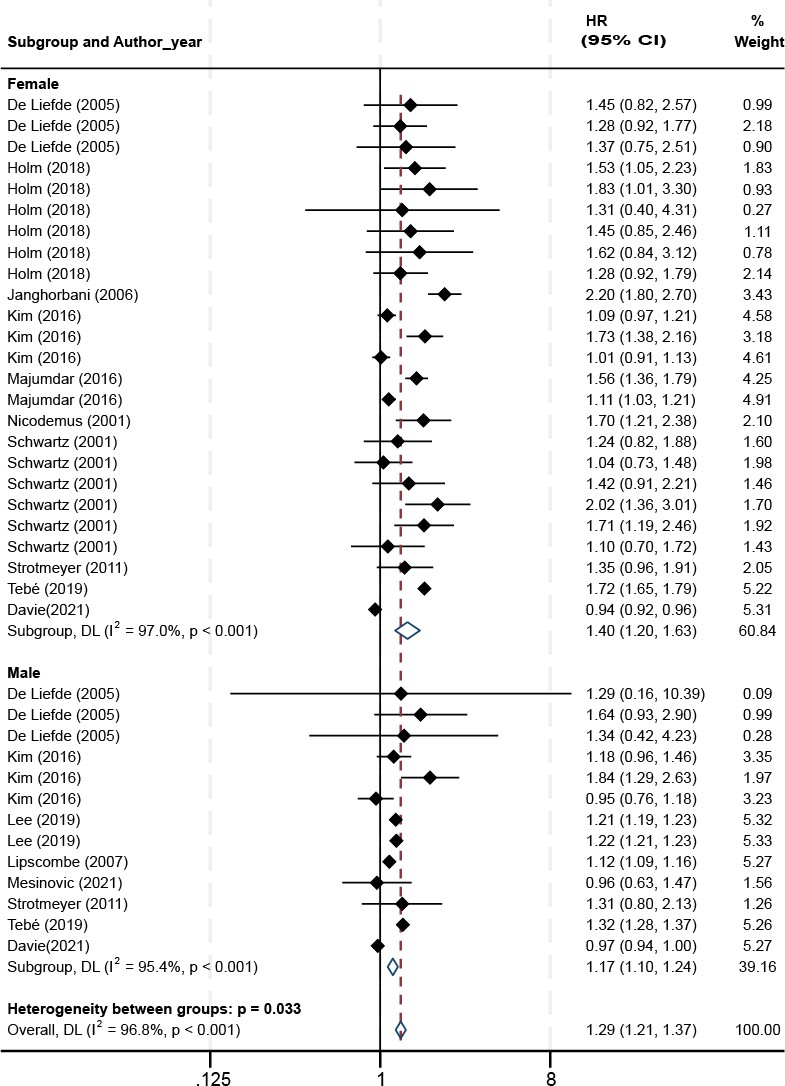


**Fig. S6: Funnel plot for publication bias, with pseudo 95% confidence interval**


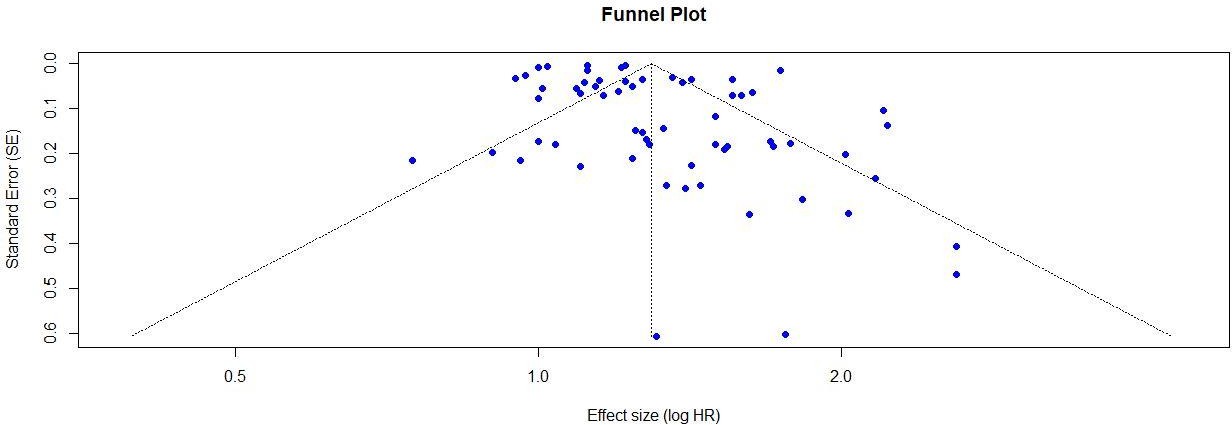

Supplement: Supplementary file 1 — Data S1: [file DME-43-e70276-s001.docx]
